# *QMrl-7B* Enhances Root System, Biomass, Nitrogen Accumulation and Yield in Bread Wheat

**DOI:** 10.3390/plants10040764

**Published:** 2021-04-13

**Authors:** Jiajia Liu, Qi Zhang, Deyuan Meng, Xiaoli Ren, Hanwen Li, Zhenqi Su, Na Zhang, Liya Zhi, Jun Ji, Junming Li, Fa Cui, Liqiang Song

**Affiliations:** 1 Center for Agricultural Resources Research, Institute of Genetics and Developmental Biology, The Innovative Academy of Seed Design, Chinese Academy of Sciences, Shijiazhuang 050022, China; ljjzl2014@163.com (J.L.); zhqiemail@163.com (Q.Z.); mengdeyuan123@163.com (D.M.); renxiaoli2011@126.com (X.R.); zhangna@sjziam.ac.cn (N.Z.); xiaoya_19861028@163.com (L.Z.); jijun@sjziam.ac.cn (J.J.); ljm@sjziam.ac.cn (J.L.); 2 University of Chinese Academy of Sciences, Beijing 100049, China; 3 College of Agronomy and Biotechnology, China Agricultural University, Beijing 100094, China; lihanwen@cau.edu.cn (H.L.); suzhenqi80@163.com (Z.S.); 4 Key Laboratory of Molecular Module-Based Breeding of High Yield and Abiotic Resistant Plants in Universities of Shandong, School of Agriculture, Ludong University, Yantai 264025, China; 5 State Key Laboratory of North China Crop Improvement and Regulation, College of Agronomy, Hebei Agricultural University, Baoding 071000, China

**Keywords:** *Triticum aestivum* L., *QMrl-7B*, root traits, grain yield, nitrogen use efficiency

## Abstract

Genetic improvement of root systems is an efficient approach to improve yield potential and nitrogen use efficiency (NUE) of crops. *QMrl-7B* was a major stable quantitative trait locus (QTL) controlling the maximum root length in wheat (*Triticum aestivum* L). Two types of near isogenic lines (A-NILs with superior and B-NILs with inferior alleles) were used to specify the effects of *QMrl-7B* on root, grain output and nitrogen-related traits under both low nitrogen (LN) and high nitrogen (HN) environments. Trials in two consecutive growing seasons showed that the root traits, including root length (RL), root area (RA) and root dry weight (RDW), of the A-NILs were higher than those of the B-NILs at seedling stage (SS) before winter, jointing stage (JS), 10 days post anthesis (PA10) and maturity (MS), respectively. Under the LN environment, in particular, all the root traits showed significant differences between the two types of NILs (*p* < 0.05). In contrast, there were no critical differences in aerial biomass and aerial N accumulation (ANA) between the two types of NILs at SS and JS stages. At PA10 stage, the aerial biomass and ANA of the A-NILs were significantly higher than those of the B-NILs under both LN and HN environments (*p* < 0.05). At MS stage, the A-NILs also exhibited significantly higher thousand-grain weight (TGW), plot grain yield, harvest index (HI), grain N accumulation (GNA), nitrogen harvest index (NHI) and nitrogen partial factor productivity (NPFP) than the B-NILs under the corresponding environments (*p* < 0.05). In summary, the *QMrl-7B* A-NILs manifested larger root systems compared to the B-NILs which is favorable to N uptake and accumulation, and eventually enhanced grain production. This research provides valuable information for genetic improvement of root traits and breeding elite wheat varieties with high yield potential and NPFP.

## 1. Introduction

Bread wheat (*Triticum aestivum* L.) is one of the major crops worldwide; its production greatly affects food security and the global economy [1]. In general, high grain productivity largely depends on water and fertilizer input. However, over-application of fertilizers has led to not only natural resources exhaustion, but also soil, air and water quality degradation [2,3]. To resolve these environmental issues and ensure food security, breeding crops with efficient use of water and nutrients is urgently required for sustainable agriculture [4]. Roots are the primary organs that determine the acquisition efficiency of soil resources and have a direct impact on grain yield [5]; more and deeper roots may improve the water and mineral uptake from deeper soil layers and reduce nitrate leaching losses to the environment [6]. Although root traits are difficult to characterize and their breeding values are seldom assessed under field conditions, manipulating root system architecture to enhance nutrient uptake has been proposed to enable a very much needed new green revolution and further increase in yield potential [7].

Root traits can be dissected into root number, root length (RL), root weight, root surface area (RA), root volume, root thickness, and density of primary roots, lateral roots and adventitious roots as well as root/shoot dry weight ratio, etc. [8,9]. Since the 1990s, a large number of quantitative trait loci (QTLs) controlling root system architecture have been reported in rice and some of them have been successfully cloned [10]. In maize, several major QTLs involving root morphology have been detected, but no causal genes have been reported yet [11]. In recent years, a good many QTLs for root traits in wheat have been also documented [9,12,13,14,15,16]. However, most of these QTLs were identified at seedling stage in hydroponic culture. It was not clear whether these root-related QTLs were associated with yield-related traits in most cases. Considering that root is mainly grown in soil and root traits are plastic in adapting to environmental factors such as limitation of water [17] and nutrients [18], field experiments under diverse environments are necessary to elucidate the genetic effects of QTLs identified in hydroponic culture. With precise evaluation and verification at the population level, QTLs associated with root traits may be used in molecular wheat breeding practice.

Nitrogen (N), as the key element of proteins and other biomacromolecules, is quantitatively the most important mineral nutrient for plant growth and development. Application of enough synthetic N fertilizers at the appropriate time can overwhelmingly improve crop yield [19]. However, only 30%~40% of the applied N fertilizer is taken up from soil by crops. Therefore, improving nitrogen use efficiency (NUE) in crops can help minimize the detrimental impact of N fertilizers on the environment and be favorable for sustainable agriculture [20,21]. As a result, a number of NUE-improved cultivars of main cereal crops have been released. Numerous research studies on rice [22,23], maize [24,25] and barley [26] indicated that root traits are closely related with N uptake and genetically controlled by major QTLs [27,28]. In wheat, several studies also discovered the co-localization of QTLs for root traits, nitrogen uptake and grain productivity [8,9]. These results presented the common genetic basis of root traits and N utilization, suggesting the tremendous potential of root traits in improving grain yield and NUE. Nevertheless, more sufficient understanding of the role of the key loci conferring high NUE will facilitate its future application in molecular breeding.

Near-isogenic lines (NILs) are powerful tools to characterize the gene/QTL function for certain plant traits [29]. We [9] detected a major stable QTL, named *QMrl-7B,* controlling the maximum root length of wheat at seedling stage in hydroponic culture and developed a pair of *QMrl-7B* NILs with superior and inferior alleles, respectively. The objective of this study was to specify *QMrl-7B*’s genetic effects on root, above-ground biomass, grain yield and nitrogen accumulation, using the pair of *QMrl-7B* NILs as materials at the population level under different nitrogen environments, which would provide a valuable resource for molecular improvement of root traits.

## 2. Results

### 2.1. Root Morphology of QMrl-7B NILs

**Figure 1:**
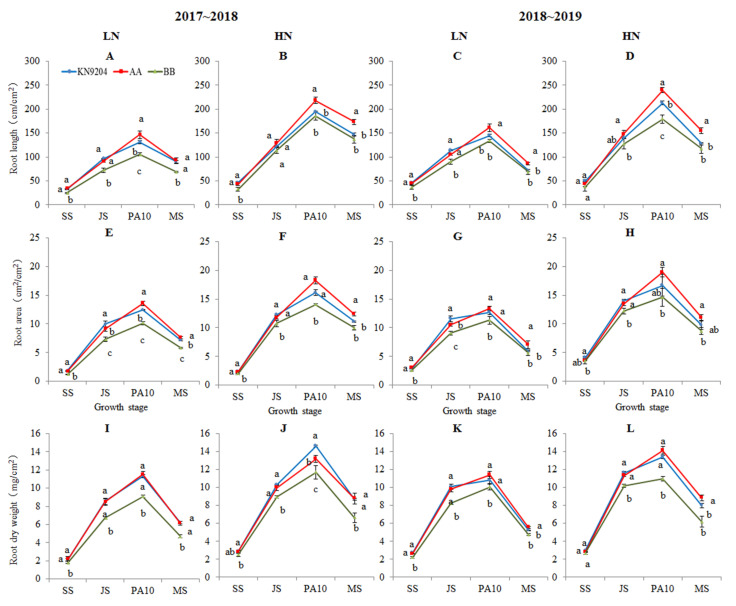
Root length (RL) (**A**–**D**), root surface area (RA) (**E**–**H**) and root dry weight (RDW) (**I**–**L**) of KN9204 and the *QMrl*-*7B* near isogenic lines (NILs) at different stages. Note: 2017~2018 and 2018~2019 indicate growing seasons; LN and HN indicate low nitrogen and high nitrogen environments, respectively; AA indicates *QMrl*-*7B* NILs with the superior alleles; BB indicates *QMrl-7B* NILs with the inferior alleles. SS, JS, PA10 and MS indicate seedling stage, jointing stage, 10 days post anthesis and maturity, respectively. Different lowercases indicate significant differences (*p* < 0.05) among the materials.

Field trials showed that the root traits of KN9204 and the *QMrl*-*7B* NILs displayed the tendency of rapid increase in the initial seedling stage and then gradual decrease with the advancement of the growth period of wheat, and the highest values of root length, root area and root dry weight of the three genotypes were recorded at the stage of 10 days post anthesis (Figure 1, Table 1). Identical changing trends in root traits were observed in both 2017~2018 and 2018~2019 growing seasons.

#### 2.1.1. Root Length (RL)

In the 2017~2018 growing season, the mean RLs of A-NILs vs. B-NILs at SS, JS, PA10 and MS were 33.6 vs. 25.1, 90.2 vs. 72.1, 146.3 vs. 105.1 and 92.2 vs. 69.0 cm/cm^2^ under the LN environment (Table 1, Figure 1A), and 42.8 vs. 32.5, 128.2 vs. 113.3, 218.4 vs. 185.6 and 173.4 vs. 137.2 cm/cm^2^ under the HN environment (Table 1, Figure 1B), respectively; indicating that RLs of the A-NILs increased 33.9%, 25.1%, 39.2% and 33.6% under LN environment, and 31.7%, 13.2%, 17.7% and 26.4% under HN environment in comparison to those of the B-NILs at the comparable stages (*p* < 0.05). In the 2018~2019 growing season, the mean RLs of the A-NILs at the comparable stages were also significantly longer than those of the B-NILs under the corresponding nitrogen environments, except the RLs at JS stage under the HN environment (Table 1; Figure 1C,D).

#### 2.1.2. Root Surface Area (RA)

In the 2017~2018 growing season, likewise, the mean RAs of A-NILs vs. B-NILs at SS, JS, PA10 and MS were 1.7 vs. 1.2, 9.1 vs. 7.3, 13.6 vs. 10.1 and 7.6 vs. 5.8 cm^2^/cm^2^ under the LN environment (Table 1, Figure 1E), and 2.3 vs. 2.0, 11.8 vs. 10.7, 18.3 vs. 14.0 and 12.3 vs. 10.0 cm^2^/cm^2^ under the HN environment (Table 1, Figure 1F), respectively; indicating that the mean RAs of the A-NILs increased by 41.7%, 24.7%, 34.7% and 31.0% under the LN environment, and 15%, 10.3%, 30.7% and 23% under the HN environment higher than those of the B-NILs at the comparable stages (*p* < 0.05). In the 2018~2019 growing season, the unvarying trends in RA difference between the two types of NILs were observed at the comparable growth stages under the corresponding nitrogen environments, except the RAs at SS stage under the HN environment (Table 1; Figure 1G,H).

#### 2.1.3. Root Dry Weight (RDW)

In the 2017~2018 growing season, similarly, the mean RDWs of A-NILs vs. B-NILs at SS, JS, PA10 and MS were 2.2 vs. 1.8, 8.5 vs. 6.7, 11.5 vs. 9.1 and 6.1 vs. 4.7 mg/cm^2^, respectively, under the LN environment (Table 1, Figure 1I), indicating that the A-NILs were heavier than the B-NILs by 22.2%, 26.9%, 26.4% and 29.8% in RDW at the four growth stages (*p* < 0.05). Under the HN environment, the mean RDWs of A-NILs vs. B-NILs at SS, JS, PA10 and MS were 2.8 vs. 2.5, 10.0 vs. 9.0, 13.2 vs. 11.7 and 8.8 vs. 6.6 mg/cm^2^, respectively (Table 1, Figure 1J), indicating that the A-NILs were 12.0%, 11.1%, 12.8% and 33.3% heavier than the B-NILs in RDW at the four growth stages (*p* < 0.05). In the 2018~2019 growing season, the mean RDWs of the A-NILs at the comparable stages were also significantly heavier than those of the B-NILs under the corresponding nitrogen environments, except the RDW at SS under the HN environment (Table 1; Figure 1K,L).

#### 2.1.4. Root Vertical Distribution

**Figure 2:**
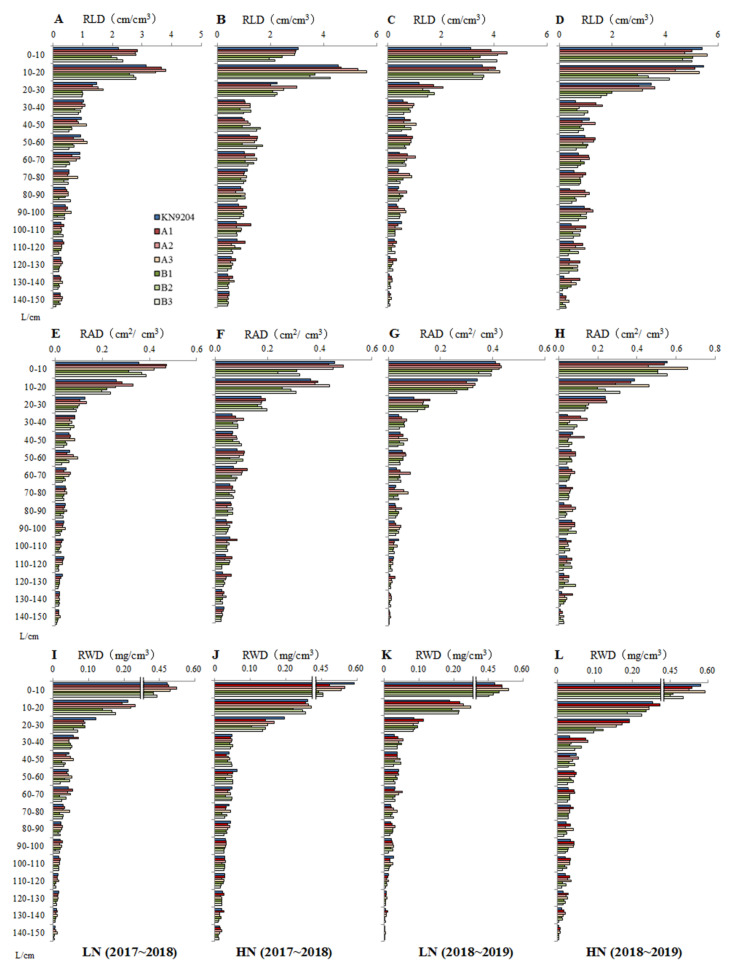
Root length density (RLD) (**A**–**D**), root area density (RAD) (**E**–**H**) and root weight density (RWD) (**I**–**L**) of KN9204 and the *QMrl*-*7B* near isogenic lines (NILs) in different soil layers at 10 days post anthesis. 2017~2018 and 2018~2019 indicate growing seasons; LN and HN indicate low nitrogen and high nitrogen environments, respectively; L indicates soil layer.

To investigate the root distribution in soil, the RLD, RAD and RWD were measured every 10 cm of soil layer at MS, JS, PA10 and MS stages. The biggest values of RLDs, RADs and RWDs at each growth stage were recorded in the upper soil layer (0~10 cm and 10~20 cm), then gradual decrease of the root indices accompanied with the raised soil depth (Figure 2, Figures S1–S3). Noticeably, the root distribution in the 30~40 cm soil layer was much less than those in the neighboring soil layers (20~30 and 40~50 cm), which may result from the restriction effect of compact soil on root growth in this ploughed bottom layer. The A-NILs exhibited superior RLDs, RADs and RWDs over the B-NILs in each soil layer (except for 30~40 cm) at the most comparable stages (*p* < 0.05). Taking the 10~20 cm soil layer at PA10 stage as an example, the mean RLDs of the A-NILs were 3.6 cm/cm^3^ in 2017~2018 and 4.0 cm/cm^3^ in 2018~2019 growing seasons under the LN environment, respectively, which were 33.3% and 14.3% higher than those of the B-NILs (2.7 and 3.5 cm/cm^3^), respectively (Figure 2A,C). Under the HN environment, the corresponding RLDs of the A-NILs were 5.2 and 4.9 cm/cm^3^, respectively, which were 36.8% and 40.0% higher than those of the B-NILs (3.8 and 3.5 cm/cm^3^) (Figure 2B,D). As expected, the RAD (Figure 2E–H) and RWD (Figure 2I–L) exhibited the consistent distribution pattern in different soil layers like RLD.

Besides, the root distribution in 0~30, 30~60, 60~100 and 100~150 cm groups of soil layers at PA10 stage was further analyzed (Table 2). The mean RL, RA and RDW of the A-NILs were significantly different from those of the B-NILs in most soil layers under the LN environment (*p* < 0.05), except for RL in the 0~30 cm soil layer and RA in the 60~100 cm soil layer in 2018~2019. Under the HN environment, significant differences in RL, RA and RDW between the two genotypes mainly took place in the 0~30 and 100~150 cm soil layers (*p* < 0.05). The ample roots of the A-NILs over the B-NILs in both upper soil and deeper soil would definitely improve water and mineral uptake, especially in water-deficient north China plain.

### 2.2. Aerial Biomass and Grain Yield of QMrl-7B NILs

#### 2.2.1. Aerial Dry Weight (ADW)

**Figure 3:**
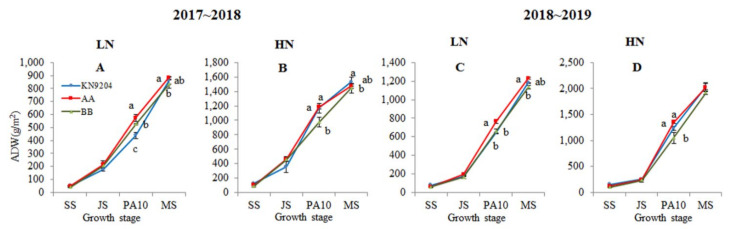
Aerial dry weight (ADW) of KN9204 and the *QMrl-7B* near isogenic lines (NILs) at different stages. 2017~2018 (**A**,**B**) and 2018~2019 (**C**,**D**) indicate growing seasons; LN (**A**,**C**) and HN (**B**,**D**) indicate low nitrogen and high nitrogen environments, respectively; AA indicates *QMrl-7B* NILs with the superior alleles; BB indicates *QMrl-7B* NILs with the inferior alleles; SS, JS, PA10 and MS indicate seedling stage, jointing stage, 10 days post anthesis and maturity, respectively; Different lowercases indicate significant differences (*p* < 0.05) among the genotypes at the same growth stage.

Field trials showed that the ADWs of KN9204 and the *QMrl*-*7B* NILs increased gradually with the advancement of wheat development (Figure 3). In the 2017~2018 growing season, the mean ADWs of A-NILs vs. B-NILs at SS, JS, PA10 and MS were 48.1 vs. 41.0, 216.0 vs. 204.1, 573.8 vs. 525.2 and 882.2 vs. 832.7 g/m^2^ under the LN environment (Figure 3A), and 100.8 vs. 89.4, 462.0 vs. 456.2, 1186.0 vs. 974.4 and 1475.2 vs. 1447.6 g/m^2^ under the HN environment, respectively (Figure 3B). In the 2018~2019 growing season, the consistent trends in ADW difference between the two types of NILs were observed repeatedly at the comparable growth stages under the corresponding nitrogen environments (Figure 3C,D).

Unlike the findings in root traits, interestingly, no significant differences in ADW were found between the two types of NILs at SS and JS stages under both LN and HN environments. The biggest difference of ADW between the two types of NILs was recorded at the stage PA10 (Figure 3). The mean ADWs of A-NILs vs. B-NILs at this stage in 2018~2019 were 762.7 vs. 658.5 g/m^2^ under the LN environment and 1349.1 vs. 1049.1 g/m^2^ under the HN environment, respectively. This finding indicated that the A-NILs were heavier than the B-NILs in ADW by 9.3% and 15.8% under the LN environment, and 21.7% and 28.6% under the HN environment in the two trial years, respectively. Prior to harvest, no significant difference between the two types of NILs in ADW was observed under the HN environment in the two growing seasons. Under the LN environment; however, the mean ADWs of A-NILs vs. B-NILs at MS were 1231.0 vs. 1135.9 g/m^2^ in 2018~2019, indicating that there were 6.76% and 8.37% phenotypic differences between the two types of NILs in the two years, respectively.

#### 2.2.2. Grain Yield

The trends of annual variation in agronomic traits of KN9204 and the *QMrl*-*7B* NILs were basically the same between the two growing seasons. Under both LN and HN environments, there were no significant differences in plant height (PH), spike length (SL), total spikelets per spike (TSPS) and kernel number per spike (KNPS) between the two types of NILs, but the A-NILs manifested superior TGW and plot grain yield over the B-NILs (Table 3). Under the LN environment, the mean TGWs of the A-NILs were 38.8 g in 2017~2018 and 40.7 g in 2018~2019, respectively, which were 1.9 g (5.15%) and 3.3 g (8.82%) heavier than those of the B-NILs (*p* < 0.05). Under the HN environment, the mean TGWs of the A-NILs were 32.6 g in 2017~2018 and 37.9 g in 2018~2019, respectively, which were 5.50% and 6.76% higher than those of the B-NILs in the comparable growing seasons (*p* < 0.05). Consequently, the A-NILs yielded more than the B-NILs. Under the LN environment, GYs of the A-NILs were 4030.9 and 5735.4 kg/ha in 2017~2018 and 2018~2019, respectively; which were 454.8 kg/ha (12.72%) and 550.2 kg/ha (10.61%) heavier than those of the B-NILs (*p* < 0.05), respectively. Under the HN environment, GYs of the A-NILs were 6388.9 and 8426.8 kg/ha in 2017~2018 and 2018~2019, respectively; which were 6.40% (6004.1 kg/ha) and 9.99% (7661.4 kg/ha) higher than those of the B-NILs (*p* < 0.05), respectively. What is more, the mean HI of the A-NILs was also significantly higher than that of the B-NILs under the corresponding nitrogen environments.

### 2.3. Nitrogen Accumulation of QMrl-7B NILs

#### 2.3.1. The Aerial N Content (ANC) and Accumulation (ANA)

**Figure 4:**
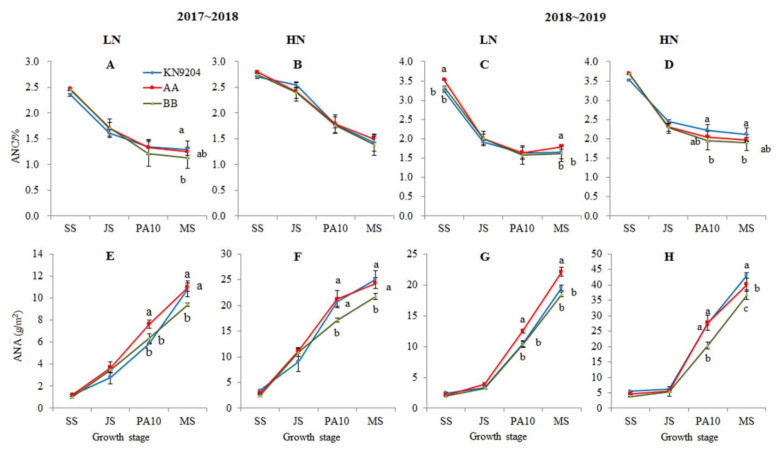
Aerial N content (ANC) (**A**–**D**) and accumulation (ANA) (**E**–**H**) of KN9204 and the *QMrl*-*7B* near isogenic lines (NILs) at different stages. 2017~2018 and 2018~2019 indicate growing seasons; LN and HN indicate low nitrogen and high nitrogen environments, respectively; AA indicates *QMrl*-*7B* NILs with the superior alleles; BB indicates *QMrl*-*7B* NILs with the inferior alleles. SS, JS, PA10 and MS indicate seedling stage, jointing stage, 10 days post anthesis and maturity, respectively; different lowercases indicate significant differences (*p* < 0.05) among the genotypes at the same growth stage.

Field trials revealed that the ANCs of KN9204 and the *QMrl*-*7B* NILs tended to decrease with the advancement of wheat development (Table S1, Figure 4A–D). The ANCs of A-NILs vs. B-NILs at SS, JS, PA10 and MS stages were 2.47% vs. 2.45%, 1.70% vs 1.71%, 1.33% vs. 1.22% and 1.25% vs. 1.13% in 2017~2018, and 3.53% vs 3.35%, 2.00% vs 2.01%, 1.64% vs. 1.57% and 1.80% vs. 1.62% in 2018~2019 under the LN environment, respectively. Under the HN environments, the ANCs of A-NILs vs. B-NILs at the comparable stages were 2.79% vs. 2.75%, 2.42% vs 2.40%, 1.79% vs. 1.76% and 1.65% vs. 1.50% in 2017~2018, and 3.69% vs 3.69%, 2.32% vs 2.30%, 2.05% vs. 1.95% and 1.98% vs. 1.91% in 2018~2019, respectively. The result showed that the A-NILs exhibited higher ANC than the B-NILs, but the differences were not significant in most cases. The significant differences were presented at SS and MS stages under the LN environment in 2018~2019 (*p* < 0.05).

The ANA tended to increase with the advancement of the growth period (Table S1, Figure 4E–H), but no significant differences were found between the two types of NILs at SS and JS. At PA10 and MS, on the other hand, the A-NILs exhibited significant higher ANA than the B-NILs under both LN and HN environments (*p* < 0.05). At PA10 stage, the mean ANAs of the A-NILs vs. B-NILs were 7.62 vs. 6.35 and 12.51 vs. 10.37 g/m^2^ under the LN environment, and 21.19 vs. 17.12 g/m^2^ and 27.69 vs. 20.36 g/m^2^ under the HN environment in the two growing seasons, respectively, indicating that the A-NILs were heavier than the B-NILs in ANA by 20.0%, 20.6%, 23.8% and 36.0% under the corresponding environments, respectively. At MS stage, the A-NILs also accumulated more N than the B-NILs, the mean ANAs of the A-NILs vs. B-NILs were 10.99 vs. 9.42 g/cm^2^ and 22.12 vs. 18.43 g/m^2^ under the LN environment, and 24.34 vs. 21.71 g/m^2^ and 40.05 vs. 36.71 g/m^2^ under the HN environment in the two growing seasons, respectively, indicating that ANAs of the A-NILs were higher than those of the B-NILs by 16.7% and 20.0% under the LN environment as well as 12.1% and 9.1% under the HN environment.

#### 2.3.2. The Grain N Content (GNC) and Accumulation (GNA)

Compared to the B-NILs, the A-NILs had higher mean GNCs, but the differences were not significant (Table 4). The GNCs of A-NILs vs. B-NILs were 2.15% vs. 2.01%, 2.64% vs. 2.44% under the LN environment, and 2.51% vs. 2.23%, and 2.98% vs. 2.87% under the HN environment in the two trial years, respectively. In contrast, there were significant differences in GNAs between the two genotypes (*p* < 0.05). The GNAs of the A-NILs vs. B-NILs were 8.9 vs. 7.2 g/m^2^ and 15.6 vs. 12.8 g/m^2^ under the LN environment, and 16.4 vs. 13.5 g/m^2^ and 25.3 vs. 22.1 g/m^2^ under the HN environment in the two years, which were 23.6%, 21.9%, 21.5%, and 14.5% higher than those of the B-NILs under the corresponding environments, respectively (Table 4).

As expected, the A-NILs manifested significant higher mean NHIs in comparison to the B-NILs under both LN and HN environments (*p* < 0.05) (Table 4). The NHIs of A-NILs vs. B-NILs were 0.81 vs. 0.77 and 0.71 vs. 0.69 under the LN environment, and 0.68 vs. 0.62 and 0.63 vs. 0.60 under the HN environment in the two consecutive growing seasons, respectively, indicating that the NHIs of the A-NILs were higher than those of the B-NILs by 2.9 to 5.2% under the LN environment and 5.0 to 9.7 under the HN environment, respectively. Meanwhile, the NPFPs of the A-NILs vs. the B-NILs were 28.02 vs. 26.33 kg kg^−1^ in 2017~2018, and 36.96 vs. 33.60 kg kg^−1^ in 2018~2019, respectively, indicating that the NPFPs of the A-NILs were 6.4% to 10.0% higher than those of the B-NILs at the normal nitrogen management (*p* < 0.05) (Table 4).

## 3. Discussion

### 3.1. The Plasticity of Wheat Root Traits Is Affected by Both Genetic and Environmental Factors

A characteristic feature of plant development plasticity is that it does not follow a rigidly predefined plan but, instead, is continuously susceptible to modification by interactions with the environment [30,31]. Root architecture is a complicated trait not only controlled by endogenous genes/QTLs but also affected by soil environment. In Arabidopsis, for example, genes such as *MONOPTEROS (MP)* and *BODENLOSBDL* regulate root architecture through repressing primary root development [32,33]. In rice, Yao et al. [34] found that the short-root mutant, *srt5*, showed extreme inhibition of seminal root, crown root and lateral root elongation, as well as altered root hair formation at the seedling stage. The PIN1 family gene, *OsPIN1* and *ZmPIN1*, plays important roles in root growth in rice [35] and maize [36], respectively. In wheat, suppression of *LATERAL ROOT DENSITY* (*LRD*) expression in RNAi plants confers the ability to maintain root growth under water limitation and has a positive pleiotropic effect on grain size and number under optimal growth conditions [37]. Overexpression of *TaTRIP1* [38]) affects the growth of root in Arabidopsis. While knockdown of the transcription factor *TabZIP60* can increase the lateral root branching in wheat [39]. Uga et al. [22] reported that the *DRO1*, a rice quantitative trait locus controlling root growth angle, is involved in cell elongation in the root tip that causes asymmetric root growth and downward bending of the root in response to gravity. Maccaferri et al. [14] revealed 20 clusters of QTLs controlling root architecture such as root length, root number and root angle of wheat. *QMrl-7B*, a major stable QTL controlling maximum root length, was reported to regulate root development of wheat in hydroponic culture of different nitrogen conditions [9]. All the above findings indicated that root architecture is mainly controlled by both major genes as well as QTLs with moderate or minor effects.

Root plastic development is enormously influenced by environmental factors including soil water deficiency [40] and insufficient nutrient availability [18]. Developmental response to drought stress in crops is manifested through enhanced root growth and suppressed shoot growth resulting in increased root/shoot ratio [41]. According to the description of Zhang et al. [17], the root growth of bread wheat in the north China plain was even before winter, remained almost static in the winter, increased rapidly between jointing and grain filling stage, and then decreased at maturity. In the present study, a pair of *QMrl-7B* near isogenic lines experienced similar root growth patterns, the root traits including root length, root surface area and root dry weight expressed plasticity to varied soil nitrogen supplies. Interestingly, there were significant differences in root traits between the two types of *QMrl-7B* NILs from seedling till mature under both low and high nitrogen environments, indicating that the *QMrl-7B* played a vital role in the maintenance of root traits (Table 1 and Table 2). *QMrl-7B* allele from KN9204 had significant positive effect on wheat root growth and development. For root vertical distribution, it was noticed that there was always significant difference between the two types of NILs, especially in deep soil, no matter what nitrogen environment there was (Table 1 and Table 2). This result further supported the permanent effect of *QMrl-7B* on root development.

### 3.2. The Association of Root System with Nitrogen Accumulation

As an integral part of plants, roots are involved in the acquisition of water and nutrients, affecting efficiency of nitrogen uptake and utilization. Several studies in maize revealed that a larger root system contributed to effective N accumulation in N-efficient cultivars in comparison with N-inefficient cultivars [42,43]. Different wheat varieties responded to low N supply by expanding their root traits, such as root length, but manifested varied N accumulation [44]. Ehdaie et al. [45] suggested that positive and significant correlation coefficients existed between root biomass and plant N content, between root biomass and grain yield in wheat. KN9204, the donor of *QMrl-7B* superior alleles, is an efficient nitrogen use wheat cultivar [46] with long roots and large root system [47]. In the current study, the two types of NILs of *QMrl-7B* did not show a significant difference in aerial nitrogen accumulation before jointing stage, but the A-NILs, with huge root systems, exhibited enhanced N accumulation in both aerial vegetative organ at anthesis and grain over the B-NILs with small root systems, particularly under LN environment (Figure 4, Table 4). These results demonstrated that *QMrl-7B* has a prolonged positive effect on N accumulation during later vegetative growth and reproductive development of wheat.

Saengwilai et al. [48] found that maize genotypes with few crown roots in six RILs had 45% greater rooting depth in low-N soils, which further enhanced N acquisition, biomass and grain yield. Li et al. [28] detected 331 QTLs for root and NUE-related traits in maize and found about 70% of QTLs for NUE-related traits co-located in a cluster with those for root traits, suggesting genetic associations between root and NUE-related traits in most cases. Some reports in wheat revealed the linkage or co-localization of root trait QTLs with N uptake QTLs [8,12]. Using the KN9204-derived RIL population, Fan et al. [9] detected a list of QTLs for root architecture and NUE-related traits, and found most of them were mapped in nine clusters. In the present study, the pleiotropic effects of *QMrl-7B* were shown by the prolonged larger root system (Figure 1 and Figure 2), higher N accumulation in the above-ground part and grain in the A-NILs (Table 3). In rice, Obara et al. [49] detected five QTLs for root system architecture and found that the most effective QTL increased the maximum root length and total root length 15.2–24.6%, in a near-isogenic line (NIL) over a wide range of nitrogen concentrations. Other studies showed that root and NUE-related traits might be regulated by the same gene. For example, overexpression of *TaNAC2-5A* enhanced root growth and nitrate influx rate in wheat, increased the root’s ability to acquire nitrogen and nitrogen accumulation in aerial parts, and eventually allocated more nitrogen in grains [50].

### 3.3. The Ideal Root System Enhances Biomass, Grain Yield and NUE

Up to now, studies principally supported the theory that larger root system is positively correlated with the enhanced nutrient uptake, biomass accumulation and yield formation [51]. In the present study, the A-NILs with superior alleles at *QMrl-7B* exhibited extremely huge root systems over the B-NILs with inferior alleles from seedling till harvest. The seedling aerial biomass of the A-NILs, interestingly, were not significantly different from those of the B-NILs (Figure 1); this insignificant difference between the two types of NILs maintained till jointing stage. At PA10 stage, the aerial biomass of the A-NILs increased dramatically and surpassed that of the B-NILs remarkably (*p* < 0.05) (Figure 1). Till mature, the root dry weight of the two types of NILs paralleled the aerial biomass and grain yield linearly. These results illustrated that there is no correlation between root biomass and the aboveground biomass in early vegetative growth of the very wheat genotype, but the huge root system formed during seedling stage potentially associates with the final biomass and grain yield. The *QMrl-7B* donor parent KN9204, as a nitrogen efficient cultivar [46], bears a larger root system, but moderate tiller number and vegetative biomass in early seedling stage compared to the well-known 1RS-1BL cultivar ‘Lovrin 10’ [47]. Comprehensively, we proposed that the luxuriant root system, rather than abundant above-ground biomass before jointing, may be essential characteristics of modern wheat cultivars with high yield and NUE.

In wheat, deep root systems contribute to greater yield potential under drought conditions [52]. The drought-adapted genotype SeriM82 showed longer root systems in deep soil layers and higher potential grain yield [41], KN9204 with its robust root system showed high grain yield and high NUE [46]. Similarly, the A-NILs with large root systems also showed a higher aerial biomass prior to harvest than the B-NILs (Figure 3), demonstrating higher potential grain yield. Some researches pointed out that abundant roots in deep soil are essential for wheat growth and final yield, especially in deficient water and nutrient stresses [53,54]. The A-NILs manifested large root systems in the 100~150 cm soil layers under both LN and HN environments, and also showed significant higher grain yield than the B-NILs. These results demonstrated that *QMrl-7B* has a positive effect on enhanced aboveground biomass and grain yield.

Among the root system architecture traits, the maximum root length decides the root depth in soil and is considered as the most important root traits to impact crop yield [55]. Cane et al. [56] detected a QTL controlling root length on chromosome 7B co-located with grain weight in durum wheat. Fan et al. [9] found the cluster C7B had striking effect on TGW and the loci *QMrl-7B* with KN9204 allele could improve TGW by 4 g (10.64%). In the present study, the mean TGW of the A-NILs was significantly higher than that of the B-NILs by 5.15% to 8.82% under the LN environment and 5.50% to 6.76% under the HN environment, respectively (Table 3), when they were planted at the population level. But the significance was much less than the effect obtained at the individual level when the RILs (10.64%) and *QMrl-7B* NILs (9.19%) were planted in a large row [9]. It seems that planting density has vital influence on the precise evaluation of the genetic effect of *QMrl-7B.* What is more, the increased TGW devoted by *QMrl-7B* greatly contributed to plot grain yield of the A-NILs, over the B-NILs by 10.61% to 12.72% under LN environment and 6.40% to 9.99% under HN environment, respectively (Table 3). These results at the population level further showed that *QMrl-7B* is of great value in elevated grain weight and grain yield.

In conclusion, NILs with superior alleles of *QMrl-7B* not only manifested a luxuriant root system, but also had positive effects on aboveground biomass, grain yield and NPFP, indicating that *QMrl-7B* could facilitate genetic improvement of wheat root system. Therefore, this study provides a valuable case that improving root system via genetic manipulation can contributes directly to increased yield and NPFP.

## 4. Materials and Methods

### 4.1. Plant Materials and Experimental Design

A major stable QTL *QMrl-7B* (controlling the maximum root length) was identified by hydroponic culture using the recombinant inbred line population derived from the cross between KN9204 and J411 (KJ-RIL) [9]. This QTL was located in the interval 89.50–92.50 cM and the candidate physical region preliminarily ranged from 580.13 to 590.13 Mb (IWGSC1.0) [9]. A residual heterozygous line KJ-RIL239, which was heterozygous within the confidence interval of *QMrl-7B* detected by twelve PCR markers across this interval [9], was selected from F_6_ progeny and self-pollinated for four generations till F_10_ progeny. Of which, two types of *QMrl-7B* NILs respectively, with superior alleles from KN9204 (A-NILs) and inferior alleles from J411 (B-NILs), were developed. In this study, the superior parent KN9204, three A-NILs (namely A1, A2 and A3) and three B-NILs (namely B1, B2 and B3) were used as materials.

The seven materials were evaluated under two different nitrogen environments in a split-plot design with three replicates at Luancheng (37°53′ N, 114°41′ E, 54 m altitude), Hebei province, China for 2017~2018 and 2018~2019 growing seasons, respectively (two years × two controlled-environments × three replicates). The low nitrogen (LN) environment was located on a long-term positioned experimental site where no nitrogen fertilizer but 600 kg ha^−1^ of superphosphate (around 16% P_2_O_5_) were applied throughout the growing period. In the high nitrogen (HN) environment, 300 kg ha^−1^ of diamine phosphate and 225 kg ha^−1^ of urea were applied before sowing, and 150 kg ha^−1^ of urea was applied at the elongation stage with irrigation every year. The field was irrigated twice at elongation and anthesis respectively to keep convenient soil hydraulic status for wheat growth. The soil fertility within the top tillage soil layer (0~20 cm) in each environment were measured after harvest (Table S2).

The plot was 6.3 m^2^ (7.0 m × 0.9 m) containing 6 rows 0.18 m apart, and 280 seeds were evenly planted in each row. All of the recommended agronomic practices were followed in each of the trials except for the nitrogen fertilization treatment as described above.

### 4.2. Root Sampling and Measurement

Roots were sampled at seedling stage before winter (SS), jointing stage (JS), 10 days post anthesis (PA10) and maturity (MS) under both LN and HN environments during the 2017~2018 and 2018~2019 growing seasons. After removing the above-ground part of the plants, the corer of 10 cm diameter was used to take the soil cores from the rows in each plot. The depth of sampling was 60, 100, 150 and 160 cm at the SS, JS, PA10 and MS at intervals of 10 cm, respectively. The soil cores were taken to the laboratory and the root samples were obtained as described by Zhang et al. [18]. The root samples were stored at –20 °C to prevent decay. On quantifying the root length (RL, cm) and root surface area (RA, cm^2^), the root samples were tiled in a transparent dish to be scanned using ScanMaker i800 Plus Scanner (600 DPI) and analyzed by LA-S software (Hangzhou Wanshen Detection Technology Co., Ltd., Hangzhou, China, www.wseen.com). After being scanned, the roots were collected, oven-dried at 105 °C for an hour and then kept at 80 °C until constant weight to determine root dry weight (RDW, mg). The root length density (RLD, cm/cm^3^), root area density (RAD, cm^2^/cm^3^) and root weight density (RWD, mg/cm^3^) were calculated using RL, RA and RDW divided by the soil core volume.

### 4.3. Yield-Related Trait Evaluation

Ten representative plants in the center of the plot were randomly sampled at physiological maturity to evaluate the yield-related traits. The plant height (PH, cm), spike number per plant (SN), spike length (SL, cm), total spikelets per spike (TSPS), sterile spikelet number per spike (SSPS), kernel number per spike (KNPS) were determined. Thousand-grain weight (TGW, g) was evaluated after harvest using the Seed Counting and Analysis System of WSeen SC-G Instrument (Hangzhou Wanshen Detection Technology Co., Ltd., Hangzhou, China, www.wseen.com). The grain yield per plot (GY, kg/ha) was measured after harvest.

### 4.4. Measurement of Nitrogen-Related Traits

Ten representative plants in each plot were randomly sampled at the stages of SS, JS, PA10 and MS, respectively, and the aerial part was oven-dried at 105 °C for an hour and then kept at 80 °C until constant weight to determine dry matter accumulation (DW, g). The aerial part at the MS was further divided into shoot and grain parts. The dry matter accumulation was corrected to the aerial dry weight per unit area (aerial dry weight, ADW, g/m^2^) and grain dry weight per unit area (grain dry weight, GDW, g/m^2^), according to the number of plants per unit area. The dried samples were ground and sifted through a 0.5 mm sieves to determine the total aerial N content (ANC, %) and total grain N content (GNC, %) using a standard Kjeldahl procedure. Based on grain yield, dry weight and total N content, a suite of traits were calculated as follows:

Harvest index (HI) = GDW/ADW

Aerial N accumulation (ANA, g/m^2^) = ANC × ADW

Grain N accumulation (GNA, g/m^2^) = GNC × GDW

N harvest index (NHI) = GNA/ANA

Partial factor productivity of applied N (NPFP, kg kg^−1^) = GY/N applied amount

Statistical analyses were conducted using the SPSS 20.0 (SPSS, Chicago, IL, United States) and the ANOVA was used to test the difference of the above traits among the genotypes at *p* < 0.05.

## Figures and Tables

**Table 1:** The RL, RA and RDW of KN9204 and the *QMrl*-*7B* NILs in different growing stages.

Root Trait	Growing Season	Material	LN				HN			
			SS	JS	PA10	MS	SS	JS	PA10	MS
RL (cm/cm^2^)	2017 ~ 2018	KN9204	32.6 ± 0.8 a	96.3 ± 0.9 a	131.0 ± 3.0 b	90.4 ± 3.0 a	46.6 ± 2.5 a	121.6 ± 0.8 a	194.5 ± 1.7 b	146.7 ± 3.2 b
		AA	33.6 ± 0.7 a	90.2 ± 1.2 a	146.3 ± 7.5 a	92.2 ± 5.7 a	42.8 ± 2.4 a	128.2 ± 8.4 a	218.4 ± 7.1 a	173.4 ± 5.1 a
		BB	25.1 ± 1.2 b	72.1 ± 4.6 b	105.1 ± 3.2 c	69.0 ± 1.6 b	32.5 ± 4.4 b	113.3 ± 6.4 b	185.6 ± 8.2 b	137.2 ± 8.2 b
	2018 ~ 2019	KN9204	46.3 ± 1.3 a	114.0 ± 1.1 a	144.7 ± 2.4 b	71.7 ± 1.8 b	49.8 ± 2.4 a	139.0 ± 0.8 ab	213.0 ± 3.6 b	127.9 ± 2.8 b
		AA	44.7 ± 3.2 a	105.4 ± 1.5 a	161.2 ± 8.4 a	86.4 ± 3.0 a	43.3 ± 5.2 a	147.9 ± 8.0 a	239.7 ± 5.7 a	154.8 ± 6.0 a
		BB	36.8 ± 3.8 b	90.2 ± 5.9 b	134.0 ± 3.2 b	68.7 ± 5.7 b	37.4 ± 8.4 b	127.4 ± 10.6 b	179.1 ± 8.5 c	117.1 ± 10.2 b
RA (cm^2^/cm^2^)	2017 ~ 2018	KN9204	1.9 ± 0.11 a	10.0 ± 0.5 a	12.4 ± 0.1 b	7.2 ± 0.19 b	2.4 ± 0.06 a	12.3 ± 0.5 a	16.1 ± 0.3 a	11.1 ± 0.11 ab
		AA	1.7 ± 0.04 a	9.1 ± 0.3 b	13.6 ± 0.4 a	7.6 ± 0.20 a	2.3 ± 0.18 a	11.8 ± 0.3 a	18.3 ± 0.4 a	12.3 ± 0.7 a
		BB	1.2 ± 0.06 b	7.3 ± 0.4 c	10.1 ± 0.3 c	5.8 ± 0.10 c	2.0 ± 0.14 b	10.7 ± 0.3 b	14.0 ± 0.7 b	10.0 ± 0.5 b
	2018 ~ 2019	KN9204	2.9 ± 0.03 a	11.6 ± 0.1 a	12.7 ± 0.5 a	5.9 ± 0.10 b	4.1 ± 0.28 a	14.0 ± 0.3 a	16.7 ± 1.9 ab	9.8 ± 0.78 ab
		AA	3.1 ± 0.20 a	10.5 ± 0.3 b	13.3 ± 0.6 a	7.1 ± 0.31 a	3.6 ± 0.07 ab	13.5 ± 0.3 a	19.0 ± 0.8 a	11.1 ± 0.5 a
		BB	2.6 ± 0.22 b	9.1 ± 0.6 c	11.3 ± 0.2 b	5.6 ± 0.40 b	3.5 ± 0.47 b	12.2 ± 0.4 b	14.6 ± 1.5 b	8.8 ± 0.61 b
RDW (mg/cm^2^)	2017 ~ 2018	KN9204	2.2 ± 0.09 a	8.5 ± 0.37 a	11.3 ± 0.2 a	6.2 ± 0.11 a	2.9 ± 0.10 a	10.3 ± 0.1 a	14.6 ± 0.1 a	8.7 ± 0.10 a
		AA	2.2 ± 0.11 a	8.5 ± 0.37 a	11.5 ± 0.3 a	6.1 ± 0.21 a	2.8 ± 0.18 ab	10.0 ± 0.31 a	13.2 ± 0.4 b	8.8 ± 0.60 a
		BB	1.8 ± 0.12 b	6.7 ± 0.13 b	9.1 ± 0.2 b	4.7 ± 0.18 b	2.5 ± 0.15 b	9.0 ± 0.15 b	11.7 ± 0.8 c	6.6 ± 0.55 b
	2018 ~ 2019	KN9204	2.7 ± 0.10 a	10.1 ± 0.28 a	10.8 ± 0.4 a	5.2 ± 0.02 b	3.0 ± 0.10 a	11.7 ± 0.13 a	13.4 ± 0.2 a	8.0 ± 0.25 b
		AA	2.6 ± 0.05 a	9.8 ± 0.28 a	11.4 ± 0.1 a	5.6 ± 0.10 a	2.8 ± 0.05 a	11.4 ± 0.15 a	14.1 ± 0.4 a	8.8 ± 0.30 a
		BB	2.2 ± 0.09 b	8.3 ± 0.15 b	10.1 ± 0.4 b	4.8 ± 0.08 b	2.7 ± 0.15 a	10.2 ± 0.18 b	11.0 ± 0.2 b	6.2 ± 0.58 c

Note: RL, RA and RDW indicate root length, root surface area and root dry weight, respectively; 2017~2018 and 2018~2019 indicate growing seasons; LN and HN indicate low nitrogen and high nitrogen environments, respectively; AA indicates *QMrl*-*7B* NILs with the superior alleles; BB indicates *QMrl*-*7B* NILs with the inferior alleles. Different lowercases indicate significant differences (*p* < 0.05) among materials at the same environment; SS, JS, PA10 and MS indicate seedling stage, jointing stage, 10 days post anthesis and maturity, respectively.

**Table 2:** The root traits of KN9204 and the *QMrl*-*7B* NILs in different soil layers at 10 days post anthesis.

Root Trait	Growing Season	Soil Layer (cm)	LN			HN			SV		
			KN9204	AA	BB	KN9204	AA	BB	E	G	E*G
RL (cm/cm^2^)	2017 ~ 2018	0~30	67.4 ± 3.1 b	79.6 ± 1.5 a	58.3 ± 3.2 c	98.2 ± 0.3 a	106.2 ± 8.6 a	81.6 ± 4.7 b	**	**	ns
		30~60	29.0 ± 1.0 a	28.9 ± 4.0 a	20.4 ± 1.8 b	31.2 ± 0.5 a	37.8 ± 1.7 a	37.5 ± 9.6 a	**	ns	*
		60~100	22.4 ± 4.4 ab	23.7 ± 3.1 a	16.4 ± 3.6 b	37.4 ± 0.8 a	42.2 ± 5.3 a	39.4 ± 4.3 a	**	ns	ns
		100~150	12.2 ± 0.6 b	14.2 ± 0.4 a	10.0 ± 1.0 c	27.6 ± 2.2 b	32.2 ± 3.4 a	27.1 ± 2.0 b	**	*	ns
	2018 ~ 2019	0~30	81.5 ± 2.6 b	98.8 ± 4 a	86.4 ± 4.8 b	142.5 ± 2.8 a	132.6 ± 6.6 a	101.7 ± 4.8 b	**	**	**
		30~60	18.2 ± 0.5 b	25.8 ± 2.1 a	21.0 ± 3.0 ab	27.2 ± 0.5 a	34.6 ± 9.2 a	26.2 ± 3.7 a	**	*	ns
		60~100	15.7 ± 5.4 b	25.6 ± 2.8 a	19.9 ± 1.7 b	26.3 ± 0.1 b	41.0 ± 4.4 a	30.2 ± 3.3 b	**	**	ns
		100~150	9.3 ± 0.1 ab	10.9 ± 2.3 a	6.7 ± 1.6 b	16.9 ± 0.2 b	31.5 ± 3.7 a	21.0 ± 5.9 b	**	**	ns
RA (cm^2^/cm^2^)	2017 ~ 2018	0~30	7.39 ± 0.5 ab	8.58 ± 0.8 a	6.60 ± 0.4 b	9.97 ± 0.3 a	10.45 ± 0.3 a	7.56 ± 0.5 b	**	**	*
		30~60	2.04 ± 0.2 a	2.15 ± 0.3 a	1.56 ± 0.3 b	2.14 ± 0.5 a	2.70 ± 0.2 a	2.49 ± 0.5 a	**	ns	ns
		60~100	1.68 ± 0.0 a	1.76 ± 0.2 a	1.23 ± 0.2 b	2.22 ± 0.4 a	2.88 ± 0.3 a	2.36 ± 0.3 a	**	**	*
		100~150	1.31 ± 0.0 a	1.11 ± 0.1 b	0.72 ± 0.1 c	1.77 ± 0.0 ab	2.22 ± 0.5 a	1.60 ± 0.2 b	**	**	*
	2018 ~ 2019	0~30	8.45 ± 0.2 ab	8.89 ± 0.1 a	7.88 ± 0.6 b	11.78 ± 0.7 a	11.39 ± 0.5 a	9.12 ± 0.8 b	**	**	ns
		30~60	1.41 ± 0.1 b	1.80 ± 0.1 a	1.50 ± 0.2 b	1.82 ± 0.2 b	2.63 ± 0.9 a	1.86 ± 0.4 b	*	ns	ns
		60~100	1.10 ± 0.4 a	1.85 ± 0.6 a	1.43 ± 0.1 a	1.80 ± 0.4 b	2.83 ± 0.3 a	1.99 ± 0.3 b	**	**	ns
		100~150	0.73 ± 0.0 ab	0.79 ± 0.2 a	0.50 ± 0.1 b	1.30 ± 0.4 b	2.16 ± 0.7 a	1.67 ± 0.8 b	**	*	ns
RDW (mg/cm^2^)	2017 ~ 2018	0~30	8.12 ± 0.1 a	8.05 ± 0.4 a	6.63 ± 0.2 b	10.42 ± 0.6 a	9.4 ± 0.5 a	8.19 ± 0.5 b	**	**	ns
		30~60	1.42 ± 0.1 ab	1.47 ± 0.1 a	1.15 ± 0.2 b	1.51 ± 0.2 a	1.29 ± 0.1 a	1.34 ± 0.2 a	ns	*	ns
		60~100	1.13 ± 0.1 a	1.32 ± 0.2 a	0.85 ± 0.1 b	1.61 ± 0.1 a	1.48 ± 0.1 ab	1.26 ± 0.2 b	**	**	ns
		100~150	0.64 ± 0.0 a	0.67 ± 0.1 a	0.45 ± 0.1 b	1.09 ± 0.0 a	1.04 ± 0.1 a	0.90 ± 0.1 b	**	**	ns
	2018 ~ 2019	0~30	7.51 ± 0.1 b	8.48 ± 0.2 a	7.70 ± 0.2 b	10.13 ± 0.5 a	9.77 ± 0.3 a	7.94 ± 0.3 b	**	**	ns
		30~60	1.09 ± 0.0 b	1.25 ± 0.1 a	1.10 ± 0.1 b	1.23 ± 0.1 a	1.55 ± 0.4 a	1.20 ± 0.3 a	ns	ns	ns
		60~100	0.91 ± 0.1 b	1.22 ± 0.2 a	0.94 ± 0.1 b	1.25 ± 0.2 b	1.59 ± 0.1 a	1.12 ± 0.1 b	**	**	ns
		100~150	0.51 ± 0.0 a	0.45 ± 0.1 a	0.32 ± 0.0 b	0.79 ± 0.0 b	1.22 ± 0.1 a	0.72 ± 0.2 b	**	**	**

Note: RL, RA and RDW indicate root length, root surface area and root dry weight, respectively; 2017~2018 and 2018~2019 indicate growing seasons; LN and HN indicate low nitrogen and high nitrogen environments, respectively; AA indicates *QMrl*-*7B* NILs with the superior alleles; BB indicates *QMrl*-*7B* NILs with the inferior alleles. Different lowercases indicate significant differences (*p* < 0.05) among materials at the same environment; SV indicate source of variation; E and G indicate environment and genotype, respectively; E*G indicate their interaction; “*” and “**” indicate significant differences at *p* < 0.05 and *p* < 0.01 levels, respectively; “ns” indicates no significant differences.

**Table 3:** Agronomic traits of KN9204 and the *QMrl*-*7B* NILs.

GS	E	Material	PH (cm)	SL (cm)	SN	TSPS	SSPS	KNPS	TGW (g)	GY (kg/ha)	HI
2017 ~ 2018	LN	KN9204	63.0 ± 3.3 b	6.6 ± 0.3 b	2.0 ± 0.0	18.1 ± 0.9 a	2.0 ± 0.8	36.9 ± 3.9 a	35.9 ± 0.7 b	3881.5 ± 19.4 b	0.46 ± 0.01 b
		AA	71.6 ± 1.4 a	7.9 ± 0.2 a	2.3 ± 0.2	15.9 ± 0.1 b	1.4 ± 0.1	33.1 ± 0.9 b	38.8 ± 0.2 a	4030.9 ± 58.9 a	0.47 ± 0.01 a
		BB	71.9 ± 1.3 a	8.0 ± 0.1 a	2.2 ± 0.1	16.2 ± 0.1 b	1.4 ± 0.2	33.2 ± 1.4 b	36.9 ± 0.3 b	3576.1 ± 76.2 c	0.43 ± 0.00 b
	HN	KN9204	67.2 ± 2.5 b	7.1 ± 0.4 b	6.4 ± 1.8	18.1 ± 1.1 a	2.1 ± 0.6	37.6 ± 5.5 a	30.1 ± 0.6 b	6851.9 ± 30.4 a	0.45 ± 0.02 a
		AA	78.0 ± 1.6 a	8.8 ± 0.2 a	6.5 ± 0.3	16.7 ± 0.3 b	1.7 ± 0.3	31.5 ± 1.4 b	32.6 ± 0.2 a	6388.9 ± 129.6 b	0.44 ± 0.01 a
		BB	76.8 ± 1.7 a	8.6 ± 0.1 a	6.3 ± 0.3	16.6 ± 0.2 b	1.8 ± 0.3	31.9 ± 0.9 b	30.9 ± 0.4 b	6004.1 ± 80.3 c	0.42 ± 0.01 b
2018 ~ 2019	LN	KN9204	70.3 ± 2.5 b	7.3 ± 0.5 b	2.8 ± 0.2	18.3 ± 0.9 a	3.4 ± 0.5	36.9 ± 1.9 a	37.8 ± 0.2 b	5257.1 ± 50.1 b	0.46 ± 0.00 b
		AA	85.0 ± 0.8 a	8.4 ± 0.2 a	3.0 ± 0.1	17.5 ± 0.3 b	2.4 ± 0.1	32.1 ± 0.9 b	40.7 ± 0.5 a	5735.4 ± 63.5 a	0.48 ± 0.01 a
		BB	83.5 ± 3.2 a	8.2 ± 0.1 a	2.9 ± 0.1	17.7 ± 0.1 b	3.0 ± 0.3	31.0 ± 1.1 b	37.4 ± 0.2 b	5185.2 ± 66.1 b	0.46 ± 0.01 b
	HN	KN9204	73.7 ± 2.5 b	8.2 ± 0.4 b	6.3 ± 2.2	19.9 ± 1.0 a	3.7 ± 0.5	36.6 ± 4.8 a	36.6 ± 0.6 ab	8054.9 ± 61.6 a	0.41 ± 0.02 ab
		AA	98.6 ± 1.3 a	8.9 ± 0.2 a	6.2 ± 0.2	19.0 ± 0.2 b	4.3 ± 0.2	29.3 ± 1.5 b	37.9 ± 0.5 a	8426.8 ± 195.2 a	0.42 ± 0.01 a
		BB	96.0 ± 0.9 a	8.5 ± 0.1 a	6.1 ± 0.1	18.4 ± 0.2 b	4.1 ± 0.5	27.7 ± 1.0 b	35.5 ± 0.5 b	7661.4 ± 244.1 b	0.40 ± 0.01 b

Note: GS indicates growing season; E indicates environment; AA indicates *QMrl*-*7B* NILs with the superior alleles; BB indicates *QMrl*-*7B* NILs with the inferior alleles; PH, SL, SN, TSPS, SSPS, KNPS, TGW, GY and HI indicate plant height, spike length, spike number, total spikelet per spike, sterile spikelet per spike, kernel number per spike, thousand-grain weight, grain yield and harvest index, respectively; different lowercases indicate significant differences (*p* < 0.05) among materials at the same environment by ANOVA.

**Table 4:** GNA, NHI and NPFP of KN9204 and the *QMrl*-*7B* NILs.

Growing Season	Environment	Material	GNC%	GNA (g/m^2^)	NHI	NPFP (kg kg^−1^)
2017~2018	LN	KN9204	2.14 ± 0.17	8.5 ± 0.3 a	0.79 ± 0.02 b	--
		AA	2.15 ± 0.05	8.9 ± 0.3 a	0.81 ± 0.01 a	--
		BB	2.01 ± 0.08	7.2 ± 0.2 b	0.77 ± 0.01 b	--
	HN	KN9204	2.33 ± 0.05	16.1 ± 0.7 a	0.64 ± 0.04 ab	30.07 ± 0.8 a
		AA	2.51 ± 0.15	16.4 ± 0.5 a	0.68 ± 0.02 a	28.02 ± 0.6 a
		BB	2.23 ± 0.05	13.5 ± 0.5 b	0.62 ± 0.03 b	26.33 ± 0.4 b
2018~2019	LN	KN9204	2.43 ± 0.06	13.3 ± 0.7 b	0.68 ± 0.00 b	--
		AA	2.64 ± 0.10	15.6 ± 0.5 a	0.71 ± 0.01 a	--
		BB	2.44 ± 0.12	12.8 ± 0.2 b	0.69 ± 0.01 b	--
	HN	KN9204	2.95 ± 0.05	24.7 ± 0.9 a	0.57 ± 0.04 b	35.30 ± 0.5 a
		AA	2.98 ± 0.04	25.3 ± 0.2 a	0.63 ± 0.02 a	36.96 ± 0.9 a
		BB	2.87 ± 0.13	22.1 ± 0.6 b	0.60 ± 0.01 b	33.60 ± 0.9 b

2017~2018 and 2018~2019 indicate growing seasons; LN and HN indicate low nitrogen and high nitrogen environments, respectively; AA indicates *QMrl*-*7B* NILs with the superior alleles; BB indicates *QMrl*-*7B* NILs with the inferior alleles; GNC indicates grain N content; GNA indicates grain N accumulation; NHI indicates N harvest index; NPFP indicates partial factor productivity of applied N; different lowercases indicate significant differences (*p* < 0.05) among the genotypes at the same environment by ANOVA.

## Supplementary Materials

The following are available online at https://www.mdpi.com/article/10.3390/plants10040764/s1, Table S1: Aerial N content (ANC) and accumulation (ANA) of KN9204 and the *QMrl-7B* NILs at different stages; Table S2: Summary of the major macronutrients in top tillage soil layer (0~20 cm) during the two growing seasons; Figure S1: Root length density (RLD), root surface area density (RAD) and root weight density (RWD) of KN9204 and the *QMrl-7B* NILs at seedling stage before winter (SS); Figure S2: Root length density (RLD), root surface area density (RAD) and root weight density (RWD) of KN9204 and the *QMrl-7B* NILs at jointing stage (JS); Figure S3: Root length density (RLD), root surface area density (RAD) and root weight density (RWD) of KN9204 and the *QMrl-7B* NILs at maturity (MS).
