# Supplementary material for: QMrl-7B Enhances Root System, Biomass, Nitrogen Accumulation and Yield in Bread Wheat
Source: Plants (Basel). 2021 Apr 13;10(4):764. doi: 10.3390/plants10040764 (PMC8069806; doi:10.3390/plants10040764)
Supplement: Supplementary file 1 [file plants-10-00764-s001.pdf]

Supplementary material

Table S1 Aerial N content (ANC) and accumulation (ANA) of KN9204 and the *QMrl-7B* NILs at different stages.

Root Trait	Growing Season	Material	LN				HN			
			SS	JS	PA10	MS	SS	JS	PA10	MS
ANC (%)	2017	KN9204	2.35±0.05 a	1.61±0.08 a	1.34±0.06 a	1.26±0.08 a	2.70±0.10 a	2.54±0.05 a	1.77±0.11 a	1.63±0.06 a
	~	AA	2.47±0.12 a	1.70±0.05 a	1.33±0.07 a	1.25±0.04 ab	2.79±0.10 a	2.42±0.06 a	1.79±0.10 a	1.65±0.05 a
	2018	BB	2.45±0.03 a	1.71±0.12 a	1.22±0.07 a	1.13±0.02 b	2.75±0.05 a	2.40±0.29 a	1.76±0.07 a	1.50±0.03 a
	2018	KN9204	3.24±0.06 b	1.91±0.08 a	1.63±0.16 a	1.65±0.03 b	3.51±0.01 a	2.44±0.05 a	2.22±0.08 a	2.11±0.02 a
	~	AA	3.53±0.10 a	2.00±0.05 a	1.64±0.02 a	1.80±0.06 a	3.69±0.12 a	2.32±0.06 a	2.05±0.14 ab	1.98±0.03 ab
	2019	BB	3.35±0.09 b	2.01±0.12 a	1.57±0.03 a	1.62±0.01 b	3.69±0.05 a	2.30±0.29 a	1.95±0.08 b	1.91±0.08 b
ANA (g/m^2)	2017	KN9204	2.11±0.02 a	2.82±0.60 a	5.85±0.04 b	10.84±0.74 a	3.44±0.14 a	9.06±1.91 a	20.62±0.86 a	25.03±1.74 a
	~	AA	1.98±0.03 a	3.68±0.50 a	7.62±0.36 a	10.99±0.38 a	2.82±0.20 a	11.16±0.54 a	21.19±1.63 a	24.34±1.12 a
	2018	BB	1.91±0.08 a	3.49±0.20 a	6.35±0.43 b	9.42±0.16 b	2.46±0.20 a	10.89±0.89 a	17.12±0.42 b	21.71±0.61 b
	2018	KN9204	2.50±0.02 a	3.35±0.29 a	10.43±1.14 b	19.52±0.22 b	5.44±0.29 a	6.13±0.14 a	27.56±0.63 a	43.05±0.84 a
	~	AA	2.15±0.07 a	3.87±0.29 a	12.51±0.31 a	22.12±0.73 a	4.57±0.15 a	5.53±0.25 a	27.69±2.43 a	40.05±2.12 b
	2019	BB	1.97±0.06 a	3.27±0.23 a	10.37±0.48 b	18.43±0.42 b	3.78±0.20 a	5.44±1.55 a	20.36±1.15 b	36.71±1.54 c

Note: 2017~2018 and 2018~2019 indicate growing seasons; LN and HN indicate low nitrogen and high nitrogen environments, respectively; AA indicates *QMrl-7B* NILs with the superior alleles; BB indicates *QMrl-7B* NILs with the inferior alleles. SS, JS, PA10 and MS indicate seedling stage, jointing stage, 10 days post anthesis and maturity, respectively; Different lowercases indicate significant differences ($p < 0.05$) among the genotypes at the same growth stage.

Table S2 Summary of the major macronutrients in top tillage soil layer (0-20 cm) during the two growing seasons

Year	Environment	Soil organic matter ($g\ kg^{-1}$)	Nitrate-N contents ($mg\ kg^{-1}$)	Total-N contents ($mg\ kg^{-1}$)	P-Olsen ($mg\ kg^{-1}$)	Available K ($mg\ kg^{-1}$)
2017-2018	LN	16.3	15.6	82.5	23.3	114.5
	HN	18.8	45.4	118.5	22.6	118.4
2018-2019	LN	16.6	16.5	85.3	23.5	115.2
	HN	18.9	46.2	122.6	23.0	116.7

Note: 2017~2018 and 2018~2019 indicate growing seasons; LN and HN indicate low nitrogen and high nitrogen environments, respectively.

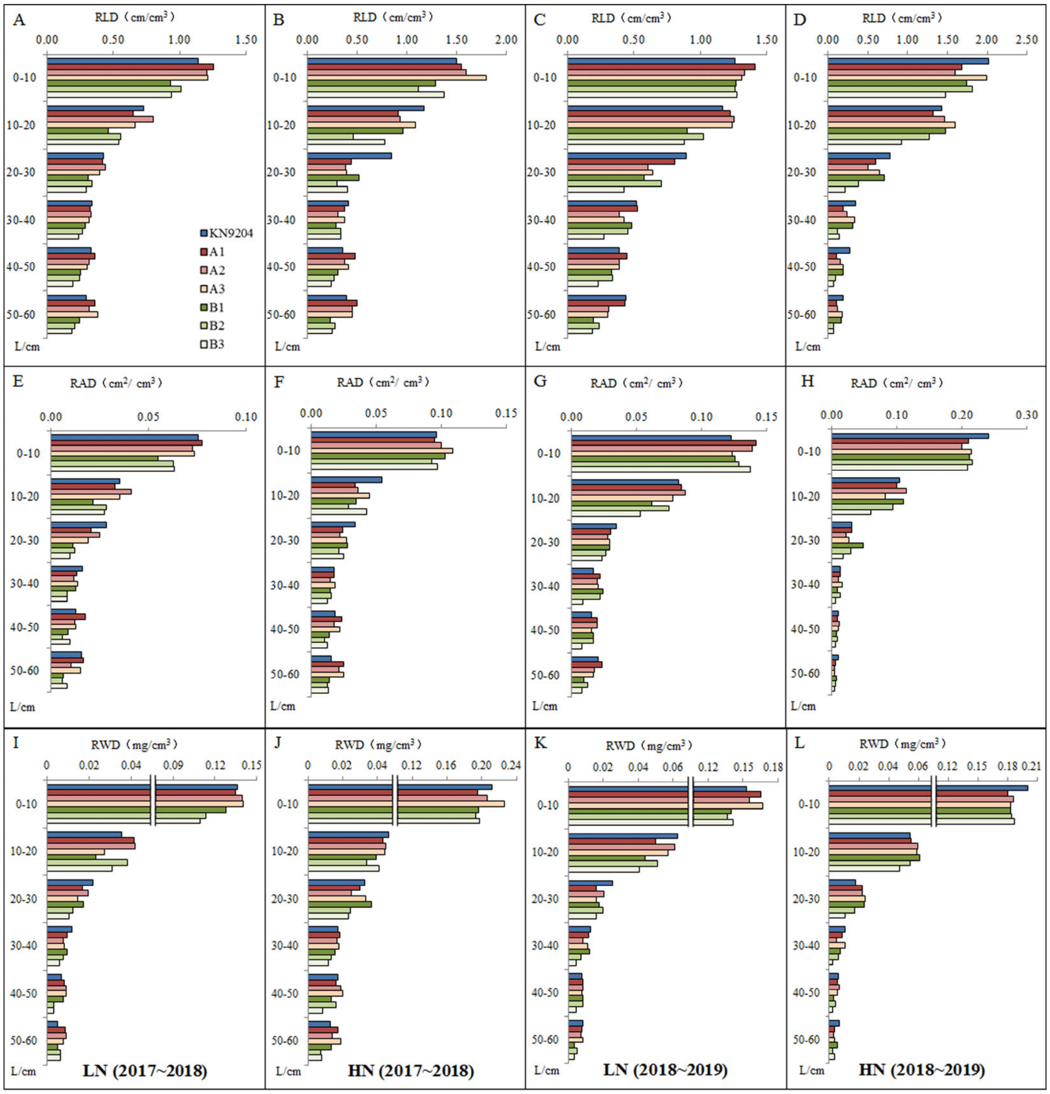

Figure S1 Root length density (RLD) (A-D), root surface area density (RAD) (G-H) and root weight density (RWD) (I-L) of KN9204 and the *QMrl-7B* NILs at seedling stage before winter (SS)

Note: 2017~2018 and 2018~2019 indicate growing seasons; LN and HN indicate low nitrogen and high nitrogen environments, respectively; L indicates soil layer.

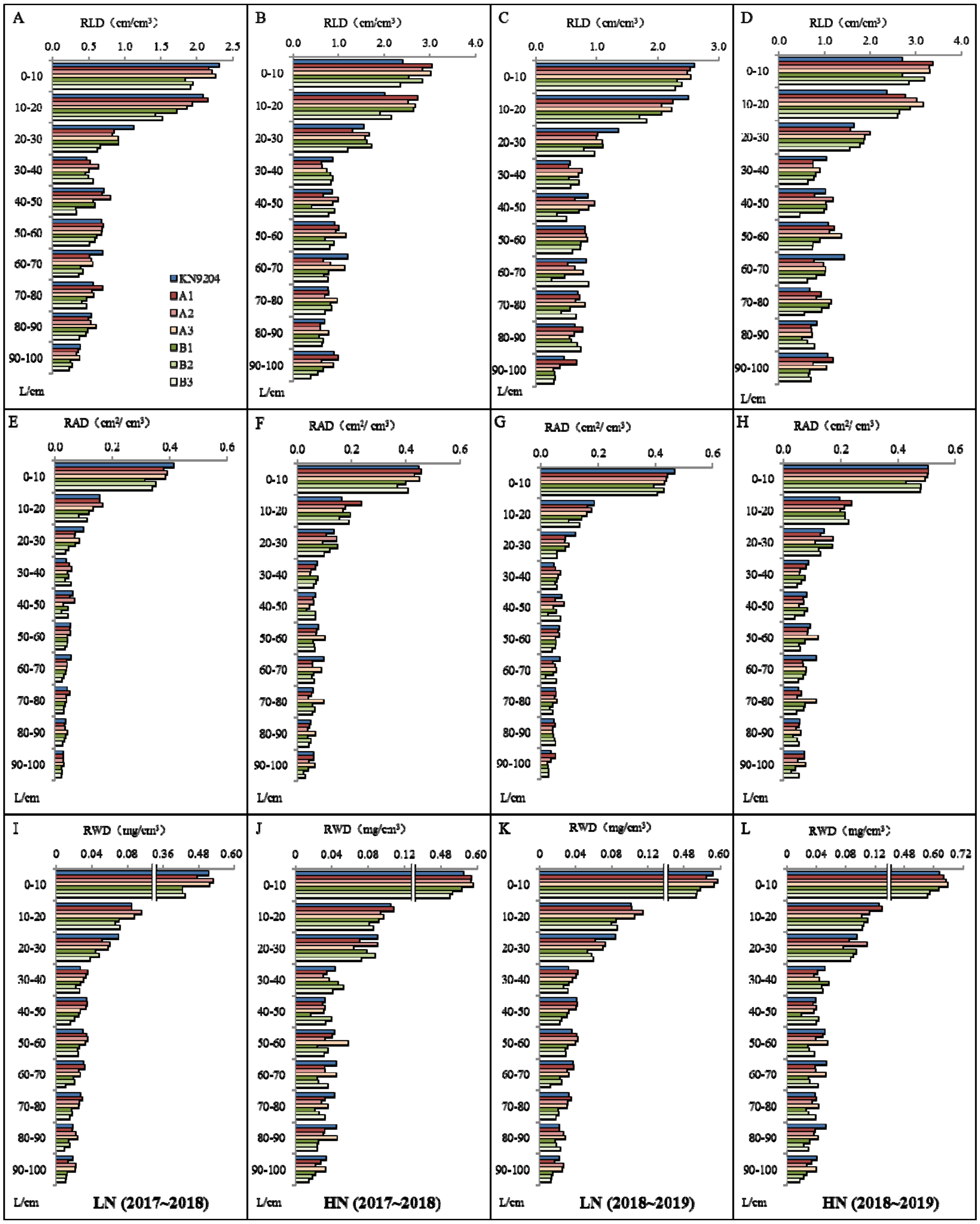

Figure S2 Root length density (RLD) (A-D), root surface area density (RAD) (E-H) and root weight density (RWD) (I-L) of KN9204 and the *QMrl-7B* NILs at jointing stage (JS)

Note: 2017~2018 and 2018~2019 indicate growing seasons; LN and HN indicate low nitrogen and high nitrogen environments, respectively; L indicates soil layer.

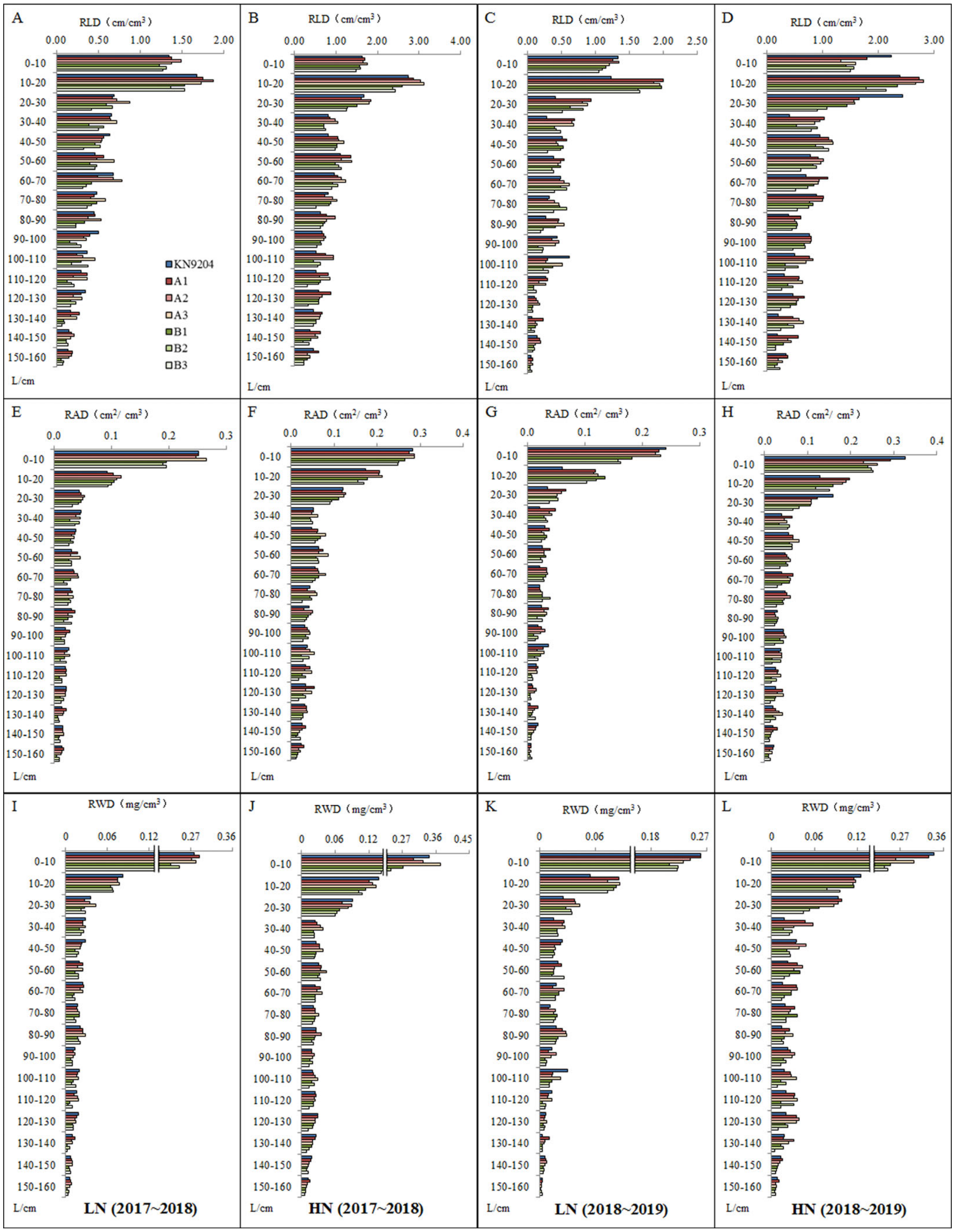

Figure S3 Root length density (RLD), root surface area density (RAD) and root weight density (RWD) of KN9204 and the *QMrl-7B* NILs at maturity (MS)

Note: 2017~2018 and 2018~2019 indicate growing seasons; LN and HN indicate low nitrogen and high nitrogen environments, respectively; L indicates soil layer.
